# Correction: Depression in the COVID-19 endemic era: Analysis of online self-disclosures by young South Koreans

**DOI:** 10.1371/journal.pone.0321589

**Published:** 2025-04-01

**Authors:** Seoyoung Kim, TaeYoon Aum, Dong-gwi Lee

The Acknowledgment section is not indicated. The Acknowledgement section is as follows: This research was supported by the National Research Foundation of Korea.
