# Supplementary material for: Depression in the COVID-19 endemic era: Analysis of online self-disclosures by young South Koreans
Source: PLoS One. 2024 Dec 26;19(12):e0314881. doi: 10.1371/journal.pone.0314881 (PMC11671000; doi:10.1371/journal.pone.0314881)
Supplement: Attachment — Submitted filename: Comments to Authors.docx [file pone.0314881.s001.docx]

This is a well-written paper on an interesting topic. Specific comments are found below.

**General comments**

- An ethics statement should be provided, given that the study involved human subjects

**Introduction**

- Can be shortened
- Explain more clearly if there were previous studies looking at the endemic blues using text-mining and what did they find

**Methods**

- Please clarify the dates used for the endemic data analysis
- Please clarify if the pandemic data and endemic data were analyzed from the same online portal
- Please explain how the endemic posts were categorized into adult/adolescence/unknown. Were the pandemic posts also categorized into adult/adolescence using the same methods?
- Are the posts all from different authors? Namely, one post per author? If not, how do the authors deal with this possible confound (e.g., many posts can be from one author, making the pool not representative of the population)? Please explain in the Methods.
- The Authors can add a “Statistics” section at the end of the Methods and include there all statistical tests conducted

**Results**

- Please add standard errors (STE) to Figures 2,3.
- Figure 3: is E6 across adults and adolescents?
