# Supplementary material for: Depression in the COVID-19 endemic era: Analysis of online self-disclosures by young South Koreans
Source: PLoS One. 2024 Dec 26;19(12):e0314881. doi: 10.1371/journal.pone.0314881 (PMC11671000; doi:10.1371/journal.pone.0314881)
Supplement: Attachment — Submitted filename: (revision) response to review comments.docx [file pone.0314881.s002.docx]

**Response to reviewers’ comments**

Depression in the COVID-19 endemic era:

Analysis of online self-disclosures by young South Koreans

| **Section** | **Review comment** | **Response** | **Page #** |
| --- | --- | --- | --- |
| General | An ethics statement should be provided, given that the study involved human subjects | Thank you for your comment on this important point. Our study did not involve human subjects. Instead, we used anonymous online posts/articles that were voluntarily and publicly shared on the Knowledge-In forum (a South Korean web forum: https://kin.naver.com/). This type of text-mining study does not require IRB approval because no procedure involves direct contact with participants. We have added an explanation on this matter on page 8 (lines 157-159) of our revised manuscript with track changes. | 8 |
| Intro. | Can be shortened | Following your suggestion, we have shortened the Introduction section by removing non-essential information and examples. | 3-8 |
|  | Explain more clearly if there were previous studies looking at the endemic blues using text-mining and what did they find | Although we could not find a previous text-mining study that focused specifically on the “endemic” blues, there were a few text-mining studies on the Corona blues. We elaborated on the previous results on page 7 (lines 124-130 of our revised manuscript with track changes).  The results showed not only that COVID-19 caused an emotional crisis to the public, but also that a collection of online texts is useful phenomenological data. | 7 |
| Methods | Please clarify the dates used for the endemic data analysis | The topic modeling data analysis was performed on March 26, 2024 (data collection: January 14, 2024). We specified each date on page 8 (line 162) and 10 (line 203) of our revised manuscript with track changes. | 8, 10 |
|  | Please clarify if the pandemic data and endemic data were analyzed from the same online portal | Yes, both data were collected/scraped from the Knowledge-In forum of the Naver portal. This explanation has now been added to page 8 (lines 162-163 of our revised manuscript with track changes).  Since we obtained the pandemic data from Bum-Hyun Kim, we used the same search terms and inclusion criteria that Kim used to collect our endemic data in order to make a fair comparison between the pandemic and endemic blues topic models. | 8 |
|  | Please explain how the endemic posts were categorized into adult/adolescence/unknown. Were the pandemic posts also categorized into adult/adolescence using the same methods? | Regarding the method we used to categorize the endemic posts into “adult”, “adolescent”, and “unknown”, we have added detailed explanations on page 10 (lines 187-196). At the same time, we acknowledge that the method is not unambiguous because the posts are from anonymous authors. This limitation is noted on page 25 (lines 487-492 of our revised manuscript with track changes).  The pandemic posts were not categorized, because our study focus was on the endemic blues. We used the pandemic posts to compare them with the endemic posts and to confirm the existence of psychological sequelae by finding topics about depression-related struggles that had persisted into the endemic era. The purpose of the categorization (for subgroup analysis within the endemic data) is now specified on page 8 (lines 146-148) to clear up the confusion. | 8,  10 |
|  | Are the posts all from different authors? Namely, one post per author? If not, how do the authors deal with this possible confound (e.g., many posts can be from one author, making the pool not representative of the population)? Please explain in the Methods. | Thank you for pointing out about the potential confounder. In an effort to control the confounder, following the means used in previous text-mining studies, we case-wise deleted all posts shared on the same day with identical title and body content to eliminate duplicates (explained on page 9, line 170 of our revised manuscript with track changes). However, we acknowledge that there is still a remaining possibility that some posts are from the same author(s). This is added as a limitation on page 26 (lines 495-503). | 26 |
|  | The Authors can add a “Statistics” section at the end of the Methods and include there all statistical tests conducted | Following your suggestion, the “Statistics” section is now added on page 12 (line 250 of our revised manuscript with track changes). The only psychological statistical method used in our study was the Mann-Whitney U test for a comparison of means between the levels of affect/sentiment expressed in the pandemic and endemic data. | 12 |
| Results | Please add standard errors (STE) to Figures 2,3. | The topic proportion estimates, or proportional changes, do not have the STE, unlike the values obtained from traditional psychological statistics. The proportional changes shown on the y-axes of Figures 2 and 3 describe the degree of variation in the topic proportion estimates at a given point in time. To resolve this confusion, we have added the definition of the y-axis to the captions of Figures 2 and 3 on page 19 (lines 349-350 and 359-360 of our revised manuscript with track changes).  A growth indicates that the topic was discussed more passionately than other topics by depressed young South Koreans at the time. The topic may decline at a later point in time as trends change. The score itself has no meaning, but the variation can reveal the trend changes. | 19 |
|  | Figure 3: is E6 across adults and adolescents? | In Figure 3, we added “predominantly among adults” next to the E6 “Social isolation due to depression and additional symptoms”, based on the topic’s tokens and a review of the raw data/original posts (as described on pages 17-18, lines 321-325 of our revised manuscript with track changes). | Fig. 3 |
